# Developing and Testing an Instrument to Measure the Factors Affecting the Salt Restriction Behaviors among Women

**DOI:** 10.34172/jrhs.2020.26

**Published:** 2020-09-30

**Authors:** Roghayeh Chenary, Akram Karimi‐Shahanjarin, Saeed Bashirian, Ghodratollah Roshanaei, Ali Akbar Fazaeli, Ali Mohammadimanesh, Mohsen Jalilian

**Affiliations:** ^1^Department of Public Health, School of Public Health, Hamadan University of Medical Sciences, Hamadan, Iran; ^2^Social Determinants of Health Research Center, Hamadan University of Medical Sciences, Hamadan, Iran; ^3^Department of Biostatistics, School of Public Health, Hamadan University of Medical Sciences, Hamadan, Iran; ^4^Modeling of Noncommunicable diseases Research center, School of Health, Hamadan University of Medical Sciences, Hamadan, Iran; ^5^Department of Health Management and Economics, School of Public Health, Tehran University of Medical Sciences, Tehran, Iran; ^6^Department of Nutrition Science, School of Medical, Hamadan University of Medical Sciences, Hamadan, Iran; ^7^Department of Public Health, School of Health, Ilam University of Medical Sciences, Ilam, Iran

**Keywords:** Eating behavior, Hypertension, Questionnaire, Psychometric, Women

## Abstract

**Background:** High salt intake is considered as one of the most important causes of hypertension and cardiovascular diseases. Measuring and identifying factors contributing to people's salt intake behaviors is important to evaluate effectiveness of interventions focusing on salt reduction behaviors. The purpose of this study was to develop and test a new theory of planned behavior (TPB)- based instrument to measure factors influencing three different salt intake behaviors (adding salt during cooking, at the table, purchasing salty food) among women.

**Study design:** A mixed-method study.

**Methods:** After the face and content validity of developed instrument were established, a representative sample of women (N= 300, age (SD):42.82(12.10)) were recruited to assess the construct validity using Partial Least Square confirmatory factor analysis. Coefficient alpha and composite reliability (CR) were used to establish reliability of instrument. The content validity index (CVI) and content validity ratio (CVR) were used to assess the content validity.

**Results:** Assessing validity and reliability of instrument led to 56-item questionnaire. CVI was more than 0.70 and CVR more than 0.56. Internal consistency as assessed by Cronbach's alpha was acceptable. Convergent and discriminant validity were established. The GOF index for behavior one was 0.250, for behavior two was 0.414 and behavior three was 0.374. The results of confirmatory factor analysis indicate that TPB model has an acceptable fit with data.

**Conclusion:** Our instrument provides a validated and reliable tool for assessing different aspects of salt intake behaviors in women to evaluate effectiveness of interventions focusing on salt reduction behaviors.

## Introduction

A healthy diet is one of the most important preventive factors fornon-communicable diseases (NCDs) including cardiovascular diseases ^1^. High salt intake is known as the main determinant of blood pressure at the individual and population levels ^2^. Moreover, there is evidence showing the association between high salt intake and stroke, left ventricular hypertrophy, the progression of kidney disease, kidney stones ^3^, osteoporosis ^4^, some types of cancers, respiratory diseases ^5^ and obesity ^6^. Therefore, salt reduction has been suggested to reduce the risk of high blood pressure and cardiovascular diseases. There is a dose-response relation between sodium reduction and blood pressure-lowering ^7^.

The mean salt intake in the world is 9 to 12 gr/day, indicating about twice recommended level ^1^. In Iran, the mean salt intake in Iran was 9.52 g/day and intake salt among about 40% of adults was at least two times higher than recommended level ^8^. In light of context and priorities, Iran developed the action plan by looking for 4.30% relative reduction in average salt intake among the population ^9^.

Population-level behavior change programs may be moderately effective in reducing salt intake. These theories inform interventions by suggesting mechanisms underlying paths between intervention components and outcomes. The added value of theoretically based interventions to reduce population salt intake has been supported by the previous studies ^10-12^. The Theory of Planned Behavior (TPB) is one of the widely used frameworks for designing dietary programs ^13-16^. This theory highlights, that human behavior is determined mainly by his/ her intention that in turn, is a function of his/her attitude, subjective norms and perceived behavioral control ^17^. Several interventions grounded in TPB have demonstrated positive outcomes in creating significant changes in reducing salt consumption ^18^.

So far, instruments have been provided to measure psychosocial determinants of salt consumption among patients with hypertension or heart disease^19,20^. As people with specific health-related conditions follow a specific diet, the uses of these instruments cannot accurately reflect the status of constructs and behavior in the general population. Thus, there is a need for a brief scale to assess psychosocial determinants of salt consumption in the community. Moreover, in some studies, several aspects of salt intake behaviors have been integrated as a whole behavior ^21^ or only one salt-related behavior has been investigated ^22^. In the field of salt intake, we identified three different behaviors including adding salt during cooking, adding salt at the table, and purchasing salty food. We believe that considering different aspects of salt intake and developing a brief scale to assess psychosocial determinants of salt intake in the community may contribute to an improved understanding of this behavior and evaluating the related interventions. Similar to many other nations, Iranian women are mainly responsible for home cooking. Therefore, the purpose of this study was to develop and test a new TPB- based instrument to measure the factors influencing salt intake behaviors in Iranian women who are responsible for family cooking.

## Methods

This study received ethics approval from Institutional Review Board of the Hamadan University of Medical Sciences.

To develop the questionnaire, we followed the guidelines described by Ajzen ^23^ and Francis et al ^24^. Applying these guidelines has been suggested to reduce the complexity faced in developing TPB's instrument ^25^. Accordingly, we used a mixed-method approach including the following steps: (a) conducting an elicitation study to extract the women's common underlying beliefs; (b) reviewing literature to identify the potential items measuring the TPB constructs related to reducing salt intake behaviors (c) conducting a cognitive interview to inform making changes in primary questionnaire and (d) conducting confirmatory factor analysis (CFA).

### Step 1: Item generation

To generate appropriate items that fit the TPB constructs, we followed both deductive and inductive methods. Accordingly, we conducted a literature review to identify survey instruments assessing TPB constructs on different behaviors related to salt intake. We also considered instruments measuring variables similar to TPB constructs under different names. Then, through a semi-structured interview (n=30), we assessed women's common underlying beliefs and thought processes of how they justify their salt intake related behaviors. We used this method to review and refine the generated items. The initial questionnaire included 81 items measuring TPB constructs of our desired behaviors (salt intake during cooking, salt in taking while eating at the table and buying salty foods). These items consisted of ten items for behavioral beliefs, ten items for outcome evaluation, 13 items for normative beliefs, 13 items for motivation to comply, ten items for control beliefs, ten items for perceived power, 12 items for intention, and three items for behaviors. These items were scored on a five-point Likert scale (one = strongly disagree, five = strongly agree).

### Step 2: Evaluate the content and face validity

The content validity of the questionnaire was evaluated using a panel of experts. The panel comprises of ten experts in the field of health education and two nutritionists. The relevancy of items was assessed using a 4-point Likert scale (1= not relevant, 2= somehow relevant, 3= quite relevant, 4= highly relevant). To ensure clarity and readability, the panel assessed the items using a 4-point Likert scale (1=not clear, 2=somehow clear, 3=clear, and 4=very clear). To calculate the CVI, the number of experts who chose options 3 and 4 was divided by the total number of experts, and values higher than 0.70 were accepted ^26^. To calculate the CVR, the panel was asked to evaluate the necessity of each item. The necessity of the items was assessed using a 3-point rating scale (1=not essential, 2=useful but not essential, 3=essential). Considering the number of experts, the CVR for scale ≥0.56 was considered satisfactory ^27^.

To assess face validity, besides testing it with the expert panel, a cognitive interview was performed. To do that, we recruited five women who fitted the criteria of potential participants to study and read the items aloud to identify their understanding of the phrasing of each item by determining the items that they were not clear or were too complex. The expert’s feedbacks and the cognitive interviews led to remove 11 items and revise in two Items, resulting in a pool of 70 items.

### Step 3: Evaluating the scale

In the third step, we conducted construct validity and reliability tests. Hence, the convergent and discriminant validity were evaluated using confirmatory factor analysis. Internal consistency reliability was evaluated using Cronbach’s alpha coefficients. Overall, 300 women were recruited and interviewed at the households' homes. Although different recommendations have been suggested to calculate the sample size and power estimates for a confirmatory factor analytic models, a common recommendation is to use the sample sizes of at least 200/5 or ten cases per parameter^28,29^. We used a cluster sampling method using a national survey sampling framework ^30^ to select women who were responsible for household cooking. Participants were asked to provide demographic data and complete the TPB questionnaire.

Ethical approval for study was provided by Hamadan University of Medical Sciences Ethical Committee (IR.UMSHA.REC.13970882).

### Data analysis

SPSS 24.0 (Chicago, IL, USA), and SmartPLS 3.2.8 were used to assess reliability and validity of constructs (convergent and divergent validity) and confirmatory factor analysis (CFA). First, KMO and Bartlett values were performed to evaluate the adequacy of sample size and sphericity of relationship before confirmatory factor analysis ^28^. Then, Questions were removed that did not achieve a factor loading of 0.4 or greater^31,32^. Cronbach's Alpha, as well as the composite and communality reliability test were used to evaluate construct reliability. We considered values greater than 0.6 as an acceptable cut-off point for Cronbach's Alpha^33,34^ and composite reliability tests^35,36^. For communality reliability test, the acceptable value for each variable was considered to be greater than 0.5.

The degree of inter-relation for items of each construct was assessed using convergent validity. To do that, the average variance extracted (AVE) and composite reliability (CR) were calculated and AVE> 0.5, and CR>AVE were considered as strong inter-relation of items. To assess the divergent validity of scale, we used the Fornel Larker test^36,37^. The quality of model was examined by the blindfolding approach, proposed by Wold^36,38^, so cv-communality and the cv-redundancy were used as an indicator for quality of measurement and structural model, respectively^37,39^. Moreover, the goodness-of-fit (GOF) was used as a model quality assessment index. To evaluate GOF, the explained variance (R^2^) was calculated. GOF values ​​are compared with three values ​​of 0.10, 0.25 and 0.36, which show poor, moderate and strong predictive quality, respectively^37,40^.

## Results

Overall, 300 women aged 42.82 ±12.10 were included in the analysis. The majority of them were married (82%), and the housewives (78%). About 38% of women reported completing high school, followed by 35% who had some university education, and 27% with less education. Only 15% of participants described their socio-economic status as high.

### Content and face validity

The first round of content validity evaluation revealed one item with CVI in clarity category less than 0.70 and ten items with a CVR less than 0.56. However, after modification, the CVI and CVR reached an acceptable level. Moreover, nine items were described as “unnecessary" by the experts and removed from the scale. Four items were merged because of semantic similarity. Moreover, some wording corrections were done in accordance with suggestions provided by experts and women. The final instrument contained 70 items including:

***Behavior 1:*** 22 items for adding salt during cooking consisted of three items for precursors of attitude behavioral beliefs and outcome evaluation, three items for precursors of subjective norms (normative beliefs and motivation to comply), three items for precursors of perceived behavioral control (control beliefs and perceived power), and three items for behavioral intention (Table 1). The behavior was measured using a single item that asked” How many times have you cooked salty food in the past week?”

**Table 1:** Scale properties, PLS measurement model and convergent validity

**Construct**	**Question**	**Factor** **loading**	**CR**	**AVE**	**Com.**	**CV.** **Com.**
**Behavioral beliefs (α = 0.656)**						
Behavior 1			0.741	0.597	0.597	0.003
A1.1	I believe that adding more salt during cooking makes it tasty.	0.620				
A1.3	I believe cooking low-salt foods makes me look like an inexperienced cook.	0.900				
Behavior 2			0.641	0.500	0.500	0.073
A2.1	I believe that adding salt at the table will increase blood pressure.	0.687				
A2.3	According to my religious belief, beginning the meal with eating some salt has health benefits.	0.662				
A2.4	According to my religious belief, beginning the meal with eating some salt increases table blessing.	0.573				
Behavior 3			0.828	0.707	0.707	0.168
A3.1	I believe that purchasing salty foods increases the risk of various diseases.	0.855				
A3.2	I believe that purchasing salty foods increases the likelihood of obesity.	0.826				
**Outcome evaluation** (**α** = **0.828)**						
Behavior 1			0.776	0.635	0.635	0.036
B1.1	Cooking a delicious food is important to me.	0.743				
B1.3	Having good cooking skills is important to me.	0.848				
Behavior 2			0.586	0.530	0.530	0.212
B2.1	preventing high blood pressure is important to me	0.737				
B2.3	Achieving health benefits from salt is important to me	0.842				
B2.4	Increasing the table blessing is important to me.	0.712				
Behavior 3			0.756	0.611	0.611	0.004
B3.1	Preventing getting diseases is important to me.	0.859				
B3.2	Preventing obesity is important to me.	0.696				
**Normative beliefs** (**α** =**0.872)**						
Behavior 1			0.757	0.512	0.512	0.096
C1.1	My husband expects me to cook salty foods.	0.806				
C1.3	My children / other people who live with me expect me to cook salty foods.	0.713				
C1.4	My guests expect me to cook salty foods.	0.615				
Behavior 2			0.727	0.500	0.500	0.025
C2.1	My husband expects me to bring salt -shaker to the table at meal times.	0.747				
C2.3	My children / other people living with me expect me to bring salt- shaker to table at meal times	0.610				
C2.4	My guests expect me to bring salt -shaker to table at meal times.	0.697				
Behavior 3			0.728	0.585	0.585	0.007
C3.3	My children / other people living with me expect me to buy salty foods.	0.575				
C3.5	TV commercials encourage me to buy salty foods.	0.916				
**Motivation to comply** (**α** = **0.666)**						
Behavior 1			0.852	0.658	0.658	0.320
D1.1	My husband’s approval of my cooking is important to me.	0.837				
D1.3	Approval of my children / other people living with me of my cooking is important to me.	0.824				
D1.4	My guests’ approval of my cooking is important to me.	0.772				
Behavior 2			0.831	0.622	0.622	0.264
D2.1	My husband’s approval of bringing salt-shaker to table at meal times is important to me.	0.836				
D2.3	Approval of my children / other people living with me of bringing salt-shaker to table is important to me.	0.792				
D2.4	My guests' approval of bringing salt-shaker to table at meal times is important to me.	0.734				
Behavior 3			0.735	0.590	0.590	0.014
D3.3	The opinion of my children / other people who live with me of my purchase behavior is important to me.	0.604				
D3.5	Buying salty foods being advertised on TV is important to me.	0.903				
**Control beliefs** (**α** =**0.663)**						
Behavior 1			0.784	0.645	0.645	0.048
E1.1	During cooking, I am tempted to add more salt because of worrying about the taste of the food.	0.620				
E1.3	When cooking some tasteless or stinky materials, I have to add more salt.	0.625				
Behavior 2			0.668	0.539	0.539	0.038
E2.1	During eating, I am tempted to add salt to make my food more delicious.	0.953				
E2.3	When eating outside the home (for example, in restaurants and parties), the salt shaker is available to add the salt.	0.410				
Behavior 3			0.705	0.500	0.500	0.009
E3.1	When shopping, I get tempted to buy salty foods.	0.782				
E3.2	TV ads are tempting me to buy salty foods.	0.619				
E3.3	The presence of a food label, facilitates buying low-salt foods.	0.590				
**Perceived power** (**α** = **0.788)**						
Behavior 1			0.830	0.710	0.710	0.177
F1.1	I can cook tasty foods without over-salting.	0.537				
F1.3	I can get rid of the bad smell and taste of some foods (such as fish) without over-salting.	0.646				
Behavior 2			0.817	0.691	0.691	0.137
F2.1	I can overcome the temptation of eating over-salted food.	0.812				
F2.3	I can avoid adding table salt, despite the availability of salt-shaker.	0.850				
Behavior 3			0.799	0.574	0.574	0.201
F3.1	I can overcome the temptation of buying salty foods.	0.843				
F3.2	I can ignore buying salty foods despite TV ads.	0.808				
F3.3	I can't ignore buying salty food despite the presence of a food label.	0.600				
**Intention** (**α** = **0.823)**						
Behavior 1			0.976	0.930	0.930	0.724
IN1.1	In the next month, I expect myself to add less salt to the food while cooking.	0.965				
IN1.2	In the next month, I want to add less salt to the food while cooking.	0.974				
IN1.3	In the next month, I intend to add less salt to the food while cooking.	0.955				
Behavior 2			0.990	0.969	0.969	0.783
IN2.1	In the next month, I expect myself to not add salt to the food at the table.	0.977				
IN2.2	In the next month, I want to not add salt to the food at the table.	0.991				
IN2.3	In the next month, I intend to not add salt to the food at the table.	0.986				
Behavior 3			0.989	0.967	0.967	0.785
IN3.1	In the next month, I expect to buy less salty foods.	0.975				
IN3.2	In the next month, I want to buy less salty foods.	0.990				
IN3.3	In the next month, I intend to buy less salty food.	0.985				

CR: composite reliability; AVE: Average variance extracted; α: Cronbach's Alpha, Com. Communality; CV. Com.: C.V. Communality

***Behavior 2:*** 26 items for adding salt at the table consisted of four items for precursors of attitude, four items for precursors of subjective norms, three items for precursors of perceived behavioral control, and three items for behavioral intention (Table 1). The behavior was measured using a single item that asked “How many times have you added salt to the food at the table in the past week? “

***Behavior 3:*** 22 items for purchasing salty foods consisted of three items for precursors of attitude, three items for precursors of subjective norms, three items for precursors of perceived behavioral control, and three items for behavioral intention (Table 1). The behavior was measured using a single item that asked “How many times have you bought salty foods in the past week? “

### KMO and Bartlett test

The Kaiser-Meyer-Olkin (KMO) measure of sampling adequacy was 0.74, 0.73, and 0.75 for behavior 1, 2 and 3, respectively. KMO values above 0.7 indicate adequate sampling^28^. Bartlett’s test of sphericity was significant (*P* <0.000) for all three behaviors which indicated the variables were not uncorrelated.

### Construct validity

**Figure 1:**
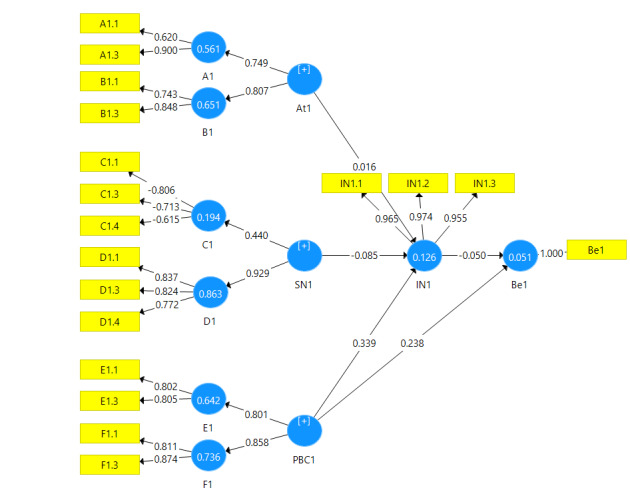
Modified measurement model in standard coefficient estimation mode (Behavior 1)

**Figure 2:**
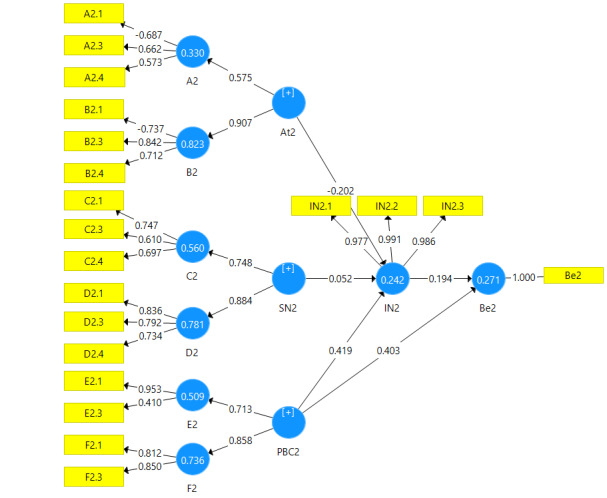
Modified measurement model in standard coefficient estimation mode (Behavior 2)

**Figure 3:**
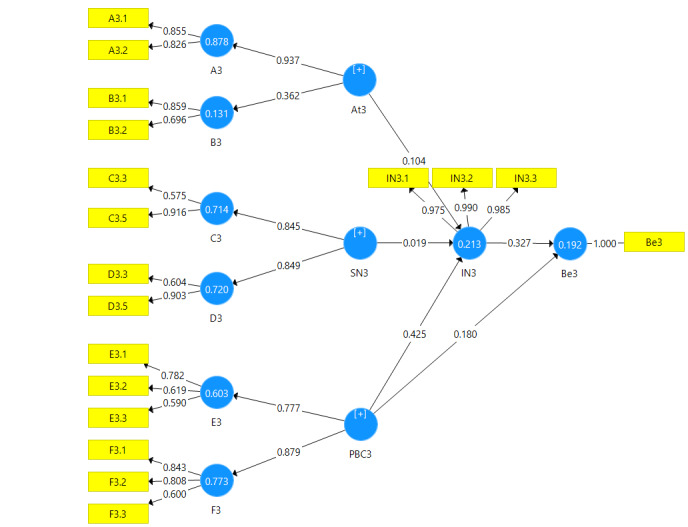
Modified measurement model in standard coefficient estimation mode (Behavior 3)

The CFA was used to evaluate how well the theoretical framework behind instrument fitted the data. Results of CFA are shown in Table 1. The measurement models fit by using CFA has been shown in Figures 1-3.

According to homogeneity test, 14 items including three items of behavioral beliefs, three items of outcome evaluation, two items of normative beliefs, two items of motivation to comply questions, two items of control beliefs question, two items of perceived power were removed due to a factor loading less than 0.4. The measurement model was modified based on the remaining items.

Table 1 shows that convergent validity was confirmed by AVE values above 0.50 for constructs of three desired behaviors. Results of Fornell and Larcker tests showed that AVE square root of each construct was greater than existing correlations with other constructs, so the discriminant validity was confirmed (Table 2). Cronbach’s alpha values ranged from 0.65 to 1.00, therefore, the internal consistency reliability of all constructs proved adequate. In addition, the values of composite reliability for all constructs were within the acceptable range (more than 0.70) (Table 1).

**Table 2:** Divergent validity test (Behavior 1, 2, and 3)

**Behavior 1**							
	**A1**	**B1**	**C1**	**D1**	**E1**	**F1**	**IN1**
**A1**	0.772						
**B1**	0.213	0.797					
**C1**	0.195	0.094	0.716				
**D1**	0.165	0.187	0.076	0.811			
**E1**	0.296	0.082	0.212	0.033	0.803		
**F1**	0.291	0.206	0.148	0.092	0.380	0.843	
**IN1**	0.084	0.091	0.156	0.031	0.173	0.384	0.965
**Behavior 2**							
	**A2**	**B2**	**C2**	**D2**	**E2**	**F2**	**IN2**
**A2**	0.707						
**B2**	0.178	0.728					
**C2**	0.115	0.077	0.707				
**D2**	0.161	0.165	0.351	0.788			
**E2**	0.024	0.021	0.139	0.130	0.734		
**F2**	0.136	0.090	0.025	0.124	0.253	0.831	
**IN2**	0.209	0.177	0.112	0.030	0.337	0.373	0.985
**Behavior 3**							
	**A3**	**B3**	**C3**	**D3**	**E3**	**F3**	**IN3**
**A3**	0.841						
**B3**	0.014	0.781					
**C3**	0.047	0.030	0.765				
**D3**	0.023	0.081	0.434	0.768			
**E3**	0.177	0.115	0.401	0.334	0.707		
**F3**	0.072	0.071	0.259	0.167	0.383	0.758	
**IN3**	0.100	0.229	0.151	0.162	0.380	0.370	0.983

The average value of cv-redundancy of the structural model for intentions was 0.17(values for intention1, intention 2, and intention 3, were 0.104, 0.220, and 0.190, respectively). This value for the structural model of behaviors was 0.15(values for behavior1, behavior 2, and behavior 3, were 0.03, 0.24, and 0.17, respectively). The obtained values indicate that the structural models were predictive^37,39^.

Our model explains a small part of the variance of intention and behavior with an average global R^2^ of 0.19 and 0.17, respectively. As PLS path modeling naturally lacks fit indices such as GFI, AGFI, and RMSEA (differently from SEM-ML) the goodness-of-fit (GOF) acts as an index for validating the model. We calculated GOF for each desired behavior by the following formula:

$$GOF=\sqrt{\bar{Communality}*\bar{R^{2}}}$$

Our results showed that the GOF index for behavior 1 was 0.250, for behavior 2 was 0.414 and for behavior 3 was 0.374. Based on guidelines suggested by Wetzels et al ^41^ GOF= 0.10, 0.25, and 0.36 should be considered as small, medium and large. Therefore, the overall predictive power of the model for behavior 1 was moderate, and for behavior 2 and behavior 3 the GOF value of the model was exceeding the large cut-off point indicating that the explaining power of the model was substantial for these behaviors.

## Discussion

This study provides preliminary evidence for validity and reliability of developed scale for identifying Iranian Women’s determinants of behaviors related to salt consumption. To the best of our knowledge, there are no tools developed specifically for assessing the psychological constructs of salt consumption behaviors in women who are responsible for cooking. The recent evidence has supported partly the improvements in reductions in salt intake; however, controversy about the effectiveness of interventions largely arises from poor measurements^42^. Despite the vast application of the TPB to investigate factors associated with eating behaviors, there have not been many attempts to develop and validate assessment tools for measuring the psychological influencing factors on salt consumption behaviors. Indeed, many studies within this area have used the TPB scale without evaluating its psychometric properties. The literature review, cognitive interview, and content validity supported idea that salt consumption has different aspects and it is necessary to assess distinct behaviors including adding salt during cooking, adding salt at the table consisted, and purchasing salty food.

Within the existing instruments, none have assessed TPB constructs for different aspects of salt consumption behaviors in general population. ME Cornélio et al developed and investigated psychometric properties of an instrument to measure TPB constructs of salt consumption behaviors among hypertension patients. They assessed the scale only using the content validity and reliability ^20^.

The unique aspects of our instrument are the measurement of psychological determinants of three different salt intake behaviors rather than an aggregated behavior, including indirect measures of TPB constructs that may help in better identifying specifically targeted points for behavior change and assessing psychometric properties using various methods, including content validity, face validity, construct validity and reliability. Our reason for developing TPB constructs related to different aspects of salt reduction behaviors was to increase sensitivity of measure to capture psychological factors affecting each of these behaviors which are not necessarily similar. We identified the participants' salient beliefs through an elicitation study cited as a valuable stage in constructing a TPB questionnaire ^24^. However, in many TPB research, this component is overlooked. The elicitation study helped to inform the scale development and reflected the key socio-psychological factors of salt intake behaviors. Although our model accounted only for 19% and 17% of the variance in intention and behavior a small part of the variance of intention and behavior, respectively, this finding is somewhat in line with those of systematic review reported by McEachan et al ^43^. Through reviewing over 200 studies, TPB constructs explained 19% of the variance in behavior and 44% of the variance in intention. One explanation for low R^2^ values for intention may be that women were very "homogeneous" as a group, especially for their attitudes. Another possible explanation may be that we splitting the salt-consumption behavior into three different sub- behaviors.

The development and psychometric testing of scale had limitations. First, employed women were under-represented and 78% of participants were housewives. Therefore, the generalizability of results to employed women is unknown. Moreover, because this scale was developed for use with a sample of the population with specific cultural food practices involving salt intake, additional studies are needed to determine the validity of this instrument in diverse populations. As with any self-reported measurement, our scale relied on participants' respondents and social desirability may have affected the response.

## Conclusion

Our scale provides a validated and reliable instrument developed based on TPB for assessing different aspects of salt intake behaviors in women. This tool could be used to evaluate the effectiveness of interventions focusing on salt reduction behaviors.

## Acknowledgements

This article is part of a Ph.D. thesis in health education and health promotion approved by Hamadan University of Medical Sciences. The authors thank all participants in this study. Ethical approval for study was provided by Hamadan University of Medical Sciences Ethical Committee whit code IR.UMSHA.REC.13970882.

## Conflict of interest

The authors declared no conflicts of interest.

## Funding

This work was supported by Hamadan University of Medical Sciences (grant number 9712218019).

## Highlights

- One feature of this instrument is to address three different behaviors related to salt intake.
- Results of this study provide a reliable and valid instrument for investigating the salt intake behavior in one Iranian population.
- This instrument would be the basis to evaluate the effectiveness of interventions addressing influencing factors of salt intake behaviors.
